# Tests for predicting complications of pre-eclampsia: A protocol for systematic reviews

**DOI:** 10.1186/1471-2393-8-38

**Published:** 2008-08-11

**Authors:** Shakila Thangaratinam, Arri Coomarasamy, Steve Sharp, Fidelma O'Mahony, Shaughn O'Brien, Khaled MK Ismail, Khalid S Khan

**Affiliations:** 1 Academic Unit of Obstetrics and Gynaecology, Birmingham Women's Hospital, Birmingham, UK; 2 Assisted Conception Unit, Guy's and St Thomas Hospital, London, UK; 3 NLH Specialist Library for ENT and Audiology, John Radcliffe Hospital, Oxford, UK; 4 Academic Unit of Obstetrics and Gynaecology, Keele University School of Medicine, University Hospital of North Staffordshire, Stoke-on-Trent, UK

## Abstract

**Background:**

Pre-eclampsia is associated with several complications. Early prediction of complications and timely management is needed for clinical care of these patients to avert fetal and maternal mortality and morbidity. There is a need to identify best testing strategies in pre eclampsia to identify the women at increased risk of complications. We aim to determine the accuracy of various tests to predict complications of pre-eclampsia by systematic quantitative reviews.

**Method:**

We performed extensive search in MEDLINE (1951–2004), EMBASE (1974–2004) and also will also include manual searches of bibliographies of primary and review articles. An initial search has revealed 19500 citations. Two reviewers will independently select studies and extract data on study characteristics, quality and accuracy. Accuracy data will be used to construct 2 × 2 tables. Data synthesis will involve assessment for heterogeneity and appropriately pooling of results to produce summary Receiver Operating Characteristics (ROC) curve and summary likelihood ratios.

**Discussion:**

This review will generate predictive information and integrate that with therapeutic effectiveness to determine the absolute benefit and harm of available therapy in reducing complications in women with pre-eclampsia.

## Background

Hypertension is a common medical complication of pregnancy, affecting about 6–8% of all pregnancies[1]. Hypertensive disorders in pregnancy consist of a group of disorders that include pre-eclampsia, latent or chronic essential hypertension, a variety of renal diseases, and transient (gestational) hypertension. The definitions used to distinguish these disorders differ, leading to uncertainty about their prevalence, natural history and response to treatment. Pre eclampsia is associated with several complications[2] and remains one of the largest single cause of maternal and fetal mortality and morbidity[3,4]. They have been reported to account for 14% of direct maternal deaths and 18% of fetal or infant deaths[3,4].

Once the diagnosis of pre-eclampsia is established, timely management is of the essence to avoid or minimise mortality and morbidity. Clinical prediction of disease complications using a combination of patients' characteristics, symptoms, physical signs and investigations all of which we consider tests, forms the basis of clinical care in these situations[5]. Therefore, there is a need for guidance regarding the best testing strategies with which to predict the development of complications in pre-eclampsia. As well as allowing clinicians to avoid unnecessary interventions in low risk groups, this would allow high-risk groups to benefit from monitoring of disease severity, use of antihypertensive therapy, administration of anticonvulsants, and antenatal corticosteroids[6,7].

## Methods

A systematic quantitative overview of studies of complications of pre-eclampsia will be conducted to obtain summary estimates of accuracy of all available tests.

The proposed methodology is in line with the guidance of the NHS Centre for Reviews and Dissemination[8] and that of the Cochrane Methods Working Group on Screening and Diagnostic tests[9]. The investigation will be carried out in the following recommended steps: (i) Question formulation, (ii) Study selection and identification, (iii) Study quality assessment, (iv) Data extraction and (v) Data synthesis. Our strategy for each of these steps will be based on a prospective protocol, which is outlined below:

## Question formulation

The tests to be considered by the review are specified in the form of structured questions in Table 1. We have generated a priority list based on importance to clinical practice using a modified Delphi survey.[10] An exhaustive list of the tests and outcomes in the prediction of pre eclampsia were sent to experts in the field. Each one of the issues were rated according to their importance to clinical practice and ranked accordingly. The review will focus on the prioritised tests obtained from the survey.

**Table 1:** Structured questions for systematic review of test accuracy studies

**Question Components**	**Tests for predicting complications of pre eclampsia**
**Population**	Pregnant women with pre eclampsia
**Tests**	
***History***	Parity; Race; Maternal age; Previous severe pre eclampsia/Eclampsia; Family history of pre eclampsia/eclampsia; Obesity; Weight gain; Pre existing hypertension, renal disease, diabetes, lupus, thrombophilia, other auto immune diseases; Multiple pregnancy; Symptoms-headache, epigastric pain, nausea, visual disturbance or combination of symptoms
***Examination***	Blood pressure; Peripheral oedema; Exaggerated tendon reflexes; Clonus; Papilloedema; Retinal changes; Oliguria; Symphysio fundal height; Oxygen saturation
***Investigations***	**Biochemical**: Serum uric acid, urine dipstick (Bedside Urinalyses) 24 hour urine protein, urinary calcium excretion, hypoalbuminaemia, microalbuminuria, fibronectin, protinuria, renal and liver function tests; **Ultrasound**: Growth, liquor volume, Doppler (uterine, umbilical artery, Middle cerebral artery, venous, uteroplacental) Bio Physical Profile; **Haematological**: Anti thrombin III, platelet count, haemoglobin, fibrinogen, thrombophilia screen, Maternal serum Alpha feto protein(MSAFP), Serum Human Chorionic Gonadotropin (HCG); Computerised Tomography; Magnetic Resonance Imaging
**Outcome**	***Maternal*** Eclampsia; Pulmonary oedema; Cerebral Haemorrhage; Hepatic, renal, haematological complications; Cardiac arrest; Abruption; Thromboembolism; stroke; psychiatric problems; Complications of labour and delivery; Maternal death; Need for hospitalisation, Day care unit visits, Use of intensive care, ventilation and dialysis
	***Fetal*** Intra uterine growth restriction; Pre maturity; Abnormal p H at birth or antenatal; Abnormal Apgar; Hypoxic Ischemic Encephalopathy; Perinatal death; Long term effect, learning disabilities, Developmental and special needs after discharge; Need for neonatal intensive care admission, mechanical ventilation and duration of hospital stay
**Study design**	**Systematic review of test accuracy studies**

## Study identification and selection

**Figure 1:**
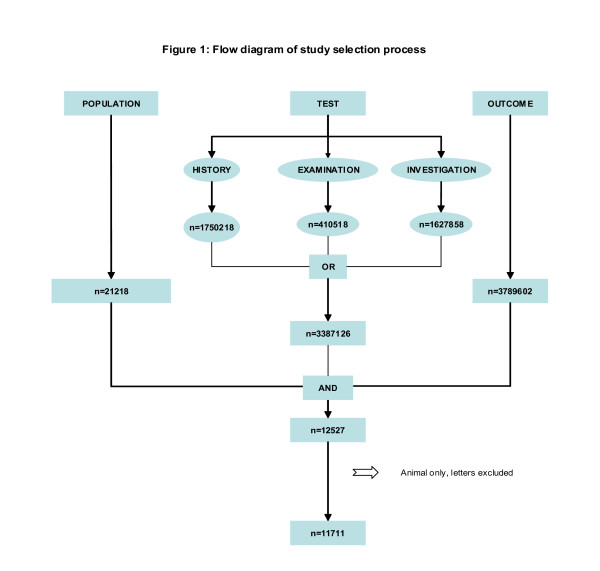
Flow diagram of study selection process in Medline.

We have a thorough search protocol by which literature is identified via general bibliographic databases including MEDLINE and EMBASE. Specialist computer databases like DARE (Database Of Abstracts of Reviews of Effectiveness), MEDION (a database of diagnostic test reviews set up by Dutch and Belgian researchers), the Cochrane Library and relevant specialist registers of the Cochrane Collaboration, particularly the Pregnancy and Child Birth group are searched. Individual experts and those with an interest in this field will be contacted to uncover grey literature. SciSearch will be used to identify frequently cited articles. Hand-searching of selected specialist journals and conference proceedings will be done to identify reports of studies for the review. Reference lists of articles obtained by iterative search will be checked as an adjunct to other methods[11]. Language restrictions will not be applied. A comprehensive database of relevant articles will be constructed. The search will be updated every year to enable inclusion of current evidence in the reviews and including other databases like SCOPUS where needed. A search term combination was constructed after exhaustive planning and piloting of possible search concepts capturing the relevant population, tests and outcomes. Our search terms and flow chart of search strategy are shown in Table 2 and in Fig 1. An initial search in Medline yielded 11711 citations. The search strategy was adapted for searching in Embase to obtain a total of 19500 citations. From this citation set, studies will be selected for inclusion in the review in a two-stage process.

**Table 2:** Search term combinations for identification of studies predicting complications of pre eclampsia

Population	Test			Outcome	
	
	History	Examination	Investigation		Final Refinement
1. pre adj eclampsia 2. preeclampsia 3. hypertens $ 4. pregnan $ 5. pre-eclampsia #.DE. 6. hypertension #.DE. 7. pregnancy #.DE. 8. 3 or 6 (hypertension) 9. 4 OR 7 (pregnancy) 10. 8 and 9 (pregnancy and hypertension)	12. history 13. parity 14. multiparity or nulliparity 15. matern$ near age 16. (previous or prior) near eclampsia 17. (previous or prior) near preeclampsia 18. (previous or prior) near pre adj eclampsia 19. multiple near pregnan$* 20. twin$ or triplet$ or quadruplet$ 21. symptom$ 22. headache 23. epigastric near pain 24. naus$ or vomit$ 25. race 26. diabet$ 27. stress 28. lupus 29. thrombophilia 30. medical-history-taking#.DE. 31. maternal-age#.DE. 32. pregnancy-multiple#.DE. 33. headache#.DE. 34. signs-and-symptoms-digestive#.DE. 35. vision-disorders#.DE. 36. weight gain#.DE. 37. population-groups#.DE. 38. diabetes-mellitus#.DE. 39. stress-psychological#.DE. 40. autoimmune-diseases#.DE. 41. thrombophilia#.DE.	43. blood adj pressure 44. oedema or edema 45. tendon$ near reflex$ 46. hyperreflexia 47. clonus 48. papilledema or papilloedema 49. retina$ near change$ 50. oliguria 51. symphys$ near fundal 52. symphys$ near height 53. cardiotocogra$ 54. oxygen near saturat$ 55. blood-pressure-determination#.DE. 56. edema#.DE. 57. reflex-abnormal#.DE. 58. retinal-diseases#.DE. 59.oliguria#.DE. 60. cardiotocography#.DE. 61.oximetry#.DE.	63. serum near uric adj acid 64. urin near analys $ 65. urin$ 66. maternal near (feto adj protein$ or fetoprotein$ or alphafetoprotein$) 67. urin$ near calcium 68. hypoalbuminemia or hypoalbuminaemia 69. microalbuminuria 70. fibronectin$ 71. proteinuria 72. renal adj function near test$ 73. liver adj function near test$ 74. liquor near volume 75. biophysical near profile 76. ultraso$ 77. antithrombin$ 78. platelet adj count 79. anti adj thrombin$ 80. fibrinogen 81. antiphospholipid $ 82. haemoglobin 83. uric-acid-QN.DE 84. alpha-fetoproteins #.DE 85. calcium-ur. DE 86. hypoalbuminemi a#.DE. 87. fibronectins .DE. 88. proteinuria #.DE. 89. kidney-function-test s#.DE. 90. liver-function-tests #.DE. 91. ultrasonography #.DE. 92. haematologic-test s#.DE. 93. antithrombin-III. DE. 94. fibrinogen #.DE. 95. antibodies-antiphospholipid #.DE. 96. diagnostic-imaging #.DE.	99. complicat$ 100. (renal or kidney$) near (disease$ or complicat$) 101. (hepatic or liver$) near (disease$ or complicat$) 102. death or mortality 103. morbidity 104. eclampsia 105. (pulmonary or lung) near (complicat$ or disease$) 106. thromboembolism 107. pulmonary near(oedema or edema) 108. ventilat$ 109. stroke 110. uter$5 near haemorrhage 111. abruption 112. (heart or cardiac) near arrest$ 113. (psychiatric or mental) near (illness$ or complication$1 or disorder) 114. hospitali$ 115. hypox$ near isch$ 116. (development$ or learning) near (disorder$ or difficult$) 117. pregnancy-complications#.DE. 118. kidney-diseases#.DE. 119. renal-dialysis# 120. liver-diseases#.DE. 121. death# 122. eclampsia# 123. pulmonary-embolism.DE. 124. respiration-artificial# 125. cerebrovascular-disorders#.DE. 126. brain-edema.DE. 127. intracranial-hypertension#.DE. 128. uterine-haemorrhage.DE. 129. abruption-placentae#.DE. 130. heart-diseases# 131. mood-disorders#.DE. 132. hospitalization# 133. infant-newborn-diseases# 134. respiratory-distress-syndrome-newborn.DE. 135. mental-disorders-diagnosed-in-childhood#.DE.	137. 11 and 98 and 136 (Captures *Population *and *Test *and *Outcome*) 138. animal = yes 139. human = yes 140. 138 not 139 141. 137 not 140 142. PT = comment or PT = letter

**11. 1 OR 2 OR 5 OR 10** **(Captures *Population***)	**42. OR/12–41** **(Captures history)**	**62. or/43–61** **(Captures examination)**	**97. or/63–96** **(Captures investigation)**	**136. or/99–135** **(Captures *Outcome***)	**143. 141 not 142** ***Final citation set (animal only studies, comments and letters excluded)***
	**98. 42 or 62 or 97 (Captures *Test***)				

**Key to commands and codes used in Dialog interface**:

Adj = words adjacent;; near = words within five words of each other in any order;.DE. = descriptor (MeSH heading);# = Exploded MeSH heading

$ = Truncated to allow for variant word endings; QN = Quick analysis pre-exploded subheading (including analysis, blood, urine, cerebrospinal fluid, isolation and purification PT = Publication Type

In the first stage the electronic searches will be scrutinised by two independent reviewers and full manuscripts of all citations that are likely to meet the predefined selection criteria will be obtained. All available reports, irrespective of language will be included to reduce bias[12]. Subsequent final inclusion or exclusion decisions will be made on examination of these manuscripts. In cases of duplicate publication, the most recent and complete versions will be selected. Two reviewers will then independently select the studies, which meet predefined and explicit criteria regarding population, tests, outcomes and study design (Table 1). These criteria will be piloted using a sample of papers and agreement between reviewers will be measured. When disagreements occur, the two reviewers will meet and if necessary the issue will be resolved by consensus involving a third reviewer.

## Study quality assessment

**Figure 2:**
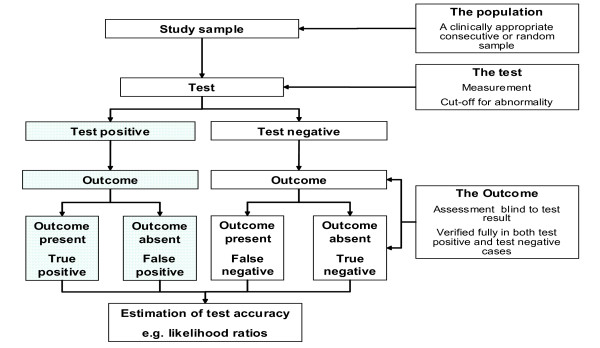
Criteria for quality assessment to be used in the review of tests predicting complications of pre eclampsia. a) Generic quality items   · Recruitment of subjects (consecutive or random sample)  · Blinding of observers assessing the outcome to the findings of the test  · Verification of diagnosis by outcome in all tested cases  · 90% or more of enrolled population followed up b) Specific quality items related to features of this project   Population  Description of spectrum composition   Test Adequate description of test and its measurements determining cut-off level for abnormality a priori  Outcome Complications of pre eclampsia

A review of papers meeting the eligibility criteria will be conducted by the same reviewers who judged eligibility, but this time rating the methodological quality of the primary research. Methodological quality is a reflection of the degree to which the study design, conduct and analysis has minimised bias in addressing the research question. This ensures a high level of internal validity (i.e. the degree to which the results of an observation are correct for the patients being studied). The potential sources of bias and variability arising from spectrum composition and other variations in test protocol or the use of reference standard in individual studies will be considered when interpreting the results[13]. In addition to using study quality as possible explanation for differences in results, the extent to which primary research met methodological standards is important per se for assessing the strength of any conclusions that are reached[8]. We will evaluate elements of study design which are likely to have a direct relationship to bias and variability in a test accuracy study [13-19]. The criteria for study validation are shown in Fig 2.

## Data extraction

The extraction of study findings will be conducted in duplicate using a pre-designed and piloted data extraction form to avoid any errors. Two authors will independently extract information from each article in order to construct 2 × 2 tables of the diagnostic test result and outcomes. Any disagreement will be resolved by consensus. Given the extent of insufficient reporting in the medical literature, we propose to obtain missing information from investigators whenever possible. It is otherwise impossible to distinguish between what was done but not reported and what was not done. To avoid introducing bias, unpublished information will be obtained in writing, and will be coded in the same fashion as published information with equal regard for inter-coder agreement. In addition to using multiple coders to insure the reproducibility of the overview, sensitivity analyses around important or questionable judgements regarding the inclusion or exclusion of studies, the validity assessments and data extraction will be performed.

## Data synthesis

We will explore causes of variation in results from study to study (heterogeneity), synthesise results from individual studies (meta-analysis) if appropriate[8,15] and assess for the risk of publication bias. Heterogeneity of results between studies will be graphically assessed looking at the distribution of rates, sensitivities and specificities in the ROC (Receiver Operating Characteristics) curve and likelihood ratios using Forest plots. To explore causes of heterogeneity we will conduct a sensitivity analysis by subgroups to see whether variations in population characteristics, tests, outcomes and study quality affect the estimate accuracy. Conclusions regarding the typical estimate accuracy will be interpreted cautiously if there is significant heterogeneity. Individual factors explaining heterogeneity will also be analysed using meta-regression to determine their unique contribution allowing for other factors. We will conduct meta-analyses to generate summary estimates of likelihood ratios (LRs), diagnostic odds ratios (ratio of LRs) and area under receiver operating characteristic (ROC) curves as appropriate[15,20,21]

The risk of publication bias is expected to be high in reviews of test accuracy[22]. Analysis for assessing the risk of publication bias will be carried out by producing funnel plots of accuracy estimates against corresponding variances. In the absence of publication bias it is to be expected that the point estimates will fill a funnel shape in the plot. Large gaps in the funnel indicate a group of possible 'missing' publications. These omissions are due to small studies showing limited accuracy and are unlikely to be missing at random. This phenomenon will also be statistically evaluated using Egger's test [23].

## Discussion

In the same way as systematic reviews of effectiveness of treatments in Obstetrics have been pursued over the last decade, research on test accuracy also needs systematic reviewing[14,24]. One of the questions remaining after establishing effectiveness evidence for magnesium sulphate, steroids and anti hypertensives is to identify those who will benefit most from these interventions[25,26]. Relying on the inclusion and exclusion criteria of the trials alone is not sufficient for determining who should and shouldn't get these treatments. Women at high risk of complications of pre-eclampsia are likely to benefit most whilst in low risk women, therapy may cause more harm than good. Therefore, what is required is the prediction of risk of complications (such as eclampsia) of pre-eclampsia.

Information on women's risk stratification can be obtained from test accuracy reviews, which provide post test probabilities for a clinical outcome targeted by treatment. Integration of these with evidence for therapeutic effectiveness will enable generating estimates of Number Needed to Treat (NNT). The lower the risk, the higher the NNT and the lower are our and women's expectation of benefit from treatment. Conversely, the higher the baseline risk, the lower the NNT, the higher are our expectation of benefit and the more inclined we would be to recommend, and women to accept therapy[24,27]. This will serve to rationalise clinical decision-making.

This project will collate and synthesise the available evidence regarding the value of the tests for predicting complications of pre-eclampsia. The systematic overviews will assess the quality of the available evidence and provide estimates of rate (or risk) of complications of pre-eclampsia given various patient characteristics and other findings. It will identify a set of tests that have maximal predictive value to aid in therapeutic decision-making. An estimate of the magnitude of the benefits will be gauged by integration of the knowledge about risk with evidence of therapeutic effectiveness for various interventions. This will help to formulate practice recommendations and specific recommendations for future research.

## Competing interests

The authors declare that they have no competing interests.

## Authors' contributions

KSK conceived the idea of the review and developed the protocol with KMKI, SO'B, FO'M, AC and ST. SS searched the electronic databases to identify the studies. KMKI, FO'M and SO'B obtained funding for ST from University Hospital North Staffordshire Research and Development Department, Stoke-on-Trent, UK. (Ref No. R 5177680). All authors read and approved the final manuscript.

## Pre-publication history

The pre-publication history for this paper can be accessed here:



## References

[B1] World Health Organization (1987). The hypertensive disorders of pregnancy.

[B2] Duvekot JJ, Peeters LLH (1994). Maternal haemodynamic adaptation to pregnancy. Obstet Gynecol Surv.

[B3] HMSO (2001). Report on Confidential Enquiries into Maternal Deaths in the United Kingdom 1997–99.

[B4] Montan SSNSN (1987). Hypertension in Pregnancy – foetal and infant outcome. Hypertension in pregnancy.

[B5] Coomarasamy A, Papaiannou S, Gee, Khan KS (2001). Aspirin to prevent pre-eclampsia in women with abnormal uterine artery doppler: a meta-analysis. Obstet Gynecol.

[B6] Duley L, Henderson-Smart DJ, Knight M, King JF (2001). Antiplatelet drugs for prevention of pre-eclampsia and its consequences: systematic review. BMJ.

[B7] Coomarasamy A, Gee H, Khan KS, Braunholtz D (2001). Aspirin has clinically significant benefit in high risk groups – Summary NNT can mislead clinicians. eBMJ.

[B8] Khan KS, Ter Riet G, Glanville J, Sowden AJ, Kleijnen J Undertaking Systematic Reviews of Research on Effectiveness. CRD's Guidance for Carrying Out or Commissioning Reviews 4 2001.

[B9] Cochrane Methods Working Group on Systematic Reviews of Screening and Diagnostic Tests: Recommended Methods. 6-6-1996.

[B10] Thangaratinam S, Ismail K, Sharp S, Coomarasamy A, O'Mahony F, Khan KS, O'Brien S (2007). Prioritisation of tests for the prediction of preeclampsia complications: a Delphi survey. Hypertension in Pregnancy.

[B11] Alderson P, Green S, Higgins JPT (2004). Cochrane Reviewer's Handbook 4.2.2.

[B12] Egger M, Zellweger-Zahner T (1997). Language bias in randomised controlled trials published in English and German. Lancet.

[B13] Whiting PJ, Rutjes AWS, Reitsma JB, Bossuyt PM, Glas AfinaS, Kleijnen J (2004). Sources of Variation and Bias in Studies of Diagnostic Accuracy A Systematic Review. Annals of Internal Medicine.

[B14] Khan KS, Dinnes J, Kleijnen J (2001). Systematic reviews to evaluate diagnostic tests. J Obstet Gynecol Reprod Biol.

[B15] Deeks JJ (2001). Systematic reviews in health care: Systematic reviews of evaluations of diagnostic and screening tests. BMJ.

[B16] Jaeschke R, Guyatt GH, Sackett DL (1994). Users' guide to the medical literature. II. How to use an article about a diagnostic test. A. Are the results of the study valid?. JAMA.

[B17] Lijmer JG, Mol BW, Heisterkamp S, Bonsel GJ, Prins MH, Meulen JHP van der (1999). Empirical evidence of design-related bias in studies of diagnostic tests. JAMA.

[B18] Bossuyt PM, Reitsma JB, Bruns DE, Glasziou PP, Gatsonis CA, Irwig LM (2004). Towards complete and accurate reporting of studies on diagnostic accuracy:The STARD initiative. Family Practice.

[B19] Chein PFW, Khan KS (2001). Evaluation of a clinical test. II: Assessment of validity. Br J Obstet Gynecol.

[B20] Jaeschke R, Guyatt GH, Sackett DL (1994). Users' guide to the medical literature. III. How to use an article about a diagnostic test. B. What are the results and will they help me in caring for my patients?. JAMA.

[B21] Honest H, Khan KS (2002). Reporting of measures of accuracy in systematic reviews in diagnostic literature. BMC Health Serv Res.

[B22] Song F, Khan KS, Dinnes J, Sutton A (2002). Asymmetric Funnel Plots and the Problem of Publication Bias in Meta-analyses of Diagnostic Accuracy. Int J Epidemiol.

[B23] Egger M, David SG, Schneider M, Minder C (1997). Bias in meta analysis detected by a simple, graphical test. BMJ.

[B24] Khan KS, Honest, Bachmann LM (2003). A generic framework for making clinical decisions integrating diagnostic and therapeutic research evidence in preterm birth. Fetal and Maternal Medicine Review. Fetal and Maternal Medicine Review.

[B25] The Magpie Trial Collaborative Group (2002). Do women with pre-eclampsia, and their babies, benefit from magnesium sulphate? The Magpie Trial: a randomised placebo-controlled trial. Lancet.

[B26] Chein PFW, Khan KS, Arnott N (1996). Magnesium sulphate in the treatment of eclampsia and pre-eclampsia: an overview of the evidence from randomised trials. Br J Obstet Gynaecol.

[B27] Coomarasamy A, Braunholtz D, Song F, Taylor R, Khan KS (2003). Individualising use of aspirin to prevent pre eclampsia: A framework for clinical decision making. BJOG.

